# Letter to the editor: Absence of cross-reactivity of the cobas WNV assay with human pegiviruses

**DOI:** 10.2807/1560-7917.ES.2025.30.21.2500316

**Published:** 2025-05-29

**Authors:** Paula Saá, Ke Chen, Ellen Ordinario

**Affiliations:** 1 Roche Molecular Systems, Pleasanton, California, United States; 2 Roche Sequencing Solutions, Pleasanton, California, United States

**Keywords:** West Nile virus, human pegivirus, blood screening, transfusion transmissible infections, virology

**To the editor:** We read with great interest the publication by Orrú et al. describing their assessment of the German risk minimisation measures implemented to prevent transfusion transmitted cases of WNV, and their evaluation of the analytical sensitivity of nucleic acid amplification technologies (NAT) used for blood screening [1]. Their study involved follow-up testing of suspected WNV-reactive blood donor samples identified during routine screening at various blood establishments, using three technologies: NAT-based PCR, metagenomic next-generation sequencing (mNGS) and serology. Confirmatory testing was performed by health authorities on samples submitted by the blood establishments with the following caveats: sample volume limitations which prevented testing of all samples with each of the three methods, and sample storage conditions which may have resulted in RNA degradation and may have impacted assay sensitivity between initial NAT-based PCR screening and follow-up mNGS testing. In addition to the limitations presented by the authors, it is important to note that the reported limit of detection (LOD) of the mNGS technology employed by Orrú et al. of 10,000 copies/mL is orders of magnitude higher than that of the cobas WNV assay, which has an LOD of 12.9 copies/mL for WNV L1 and 6.2 copies/mL for WNV L2 [1,2]. It is therefore possible that follow-up testing of samples that were stored under suboptimal conditions, using a technology ca 1,000 X less sensitive than the cobas WNV assay may have failed to detect low levels of WNV RNA present in blood. In fact, according to the authors, 28 of the 54 donations that were reactive on the cobas WNV assay performed by health authorities had viral loads lower than 200 IU/mL or 94 copies/mL, which are significantly below the LOD of the mNGS assay. It is also highly possible that this was the case for the remaining 17 samples containing viral loads > 200 IU/mL or > 94 copies/mL.

One surprising finding from this study was that mNGS testing of suspected WNV-reactive blood samples identified two cases of human pegivirus type-2 (HPgV-2), leading the authors to suggest that the WNV-NAT methods in use for blood screening in Germany, which include the cobas WNV assay, might have broad cross-reactivity among other flaviviruses beyond the JEV serogroup. To investigate this finding, our group performed in silico analysis by mapping the cobas WNV assay target oligonucleotides to all HPgV genome sequences available in the NCBI database and observed low sequence similarity for all oligonucleotides, which does not support cobas WNV assay cross-reactivity with HPgV-2. Moreover, alignment of full length WNV lineages 1 and 2 genomes with HPgV-2 showed less than 40% overall homology. Pegivirus viraemia has been previously reported in blood donor populations, with a global frequency of 1.7% and a regional frequency of 2.3% among European blood donors, and it could reach higher rates in specific locations, like Brazil with 12.42% of positive donors [3,4]. Considering the in silico and genomic analyses, as well as the differences in assay sensitivities, a plausible explanation to the identification of HPgV-2 by mNGS may be that this finding reflects the circulation of HPgV-2 in German blood donors and not cross-reactivity of the cobas WNV assay. It is important to note that target sequences of commercial assays, such as the cobas WNV assay, are not published and hence are unknown to end users. Thus, in silico analyses like those described in this letter, could not have been done by the authors of the article to support the conclusions in their manuscript.

## References

[1] Orru’ S Reissinger A Filomena A Heitmann A Funk MB Schmidt-Chanasit J Assessment of the effectiveness of West Nile virus screening by analysing suspected positive donations among blood donors, Germany, 2020 to 2023. Euro Surveill. 2025;30(8):2400373. 10.2807/1560-7917.ES.2025.30.8.2400373 40017391 PMC11869365

[2] Stanley J AuBuchon JP Erickson Y Waxman DA Williamson PC Bertuzis R Evaluation of a new West Nile virus nucleic acid test for screening of blood donations. Transfusion. 2019;59(2):623-8. 10.1111/trf.15022 30427542 PMC7379961

[3] Silva ASN Silva CP Barata RR da Silva PVR Monteiro PDJ Lamarão L Human pegivirus (HPgV, GBV-C) RNA in volunteer blood donors from a public hemotherapy service in Northern Brazil. Virol J. 2020;17(1):153. 10.1186/s12985-020-01427-6 33054824 PMC7556973

[4] Yang N Dai R Zhang X . Global prevalence of human pegivirus-1 in healthy volunteer blood donors: a systematic review and meta-analysis. Vox Sang. 2020;115(3):107-19. 10.1111/vox.12876 31845353

